# Exploring an AI-driven dynamic triage system for real-time patient risk reassessment in emergency departments in low-resource settings

**DOI:** 10.3389/fdgth.2026.1776341

**Published:** 2026-07-01

**Authors:** Kelechi John Nwadinigwe, Malik Olatunde Oduoye, Minal Jehan, Areeba Shahid, Areeb Hassan, Syeda Sidra Mudassir, Umair Bashir

**Affiliations:** 1 Emergency Medicine, Chukwuemeka Odumegwu Ojukwu University Teaching Hospital, Awka, Nigeria; 2 Department of Research, the Medical Research Circle, Goma, Democratic Republic of Congo; 3 Department of Medicine, Karachi Medical and Dental College, Karachi, Pakistan; 4 Department of Medicine and Surgery, Jinnah Sindh Medical University, Karachi, Pakistan; 5 Department of Medicine, Chandka Medical College, SMBBMU, Larkana, Sindh, Pakistan

**Keywords:** artificial intelligence, emergency medicine, low-resource settings, machine learning, triage

## Abstract

**Background:**

Emergency department (ED) overcrowding is a global challenge, particularly acute in low-resource settings, where staff and equipment shortages exacerbate inefficiencies. Conventional triage systems are static and often fail to detect patient deterioration after the initial assessment. This study aims to explore the potential of AI-driven dynamic triage systems for continuous reassessment of patient risk in low-resource EDs.

**Methods:**

A narrative review was conducted through a literature search from 2014 to 2025, following a structured selection process using PubMed, Google Scholar, Web of Science, and ResearchGate, synthesizing evidence on conventional triage models, AI- based decision support, and machine learning applications in emergency care.

**Results:**

This study shows that the Emergency Severity Index (ESI) achieves a pooled sensitivity of 81.8% and specificity of 70.5–81.7 for predicting short-term mortality and ICU admissions; yet, under-triage rates in LMICs reach 25%–30%. AI-driven models, including logistic regression, random forests, gradient boosting, and LSTM networks, demonstrate superior predictive accuracy, reducing prioritization errors by up to 9% and improving detection of high-risk patients. Pilot programs in LMICs confirm the feasibility of mobile and cloud-based AI tools, though challenges remain in data quality, infrastructure, and clinician trust.

**Conclusion:**

AI-enabled dynamic triage offers promise for enhancing patient safety, optimizing resource allocation, and reducing mortality in overcrowded EDs. However, successful implementation requires prospective validation, infrastructure investment, and co-design with clinicians to ensure adaptability in low-resource settings**.**

## Highlights

- Emergency department overcrowding is a critical global issue, especially in low-resource settings where shortages of staff and equipment intensify inefficiencies.
- Conventional triage systems are static and often fail to detect patient deterioration, with under-triage rates reaching 25%–30% in LMICs.
- AI-driven dynamic triage enables continuous patient risk reassessment, improving detection of high-risk cases and reducing prioritization errors.
- Machine learning models such as logistic regression, random forests, gradient boosting, and LSTM networks demonstrate superior predictive accuracy compared to traditional methods.
- Successful implementation of AI-enabled triage requires prospective validation, infrastructure investment, and clinician co-design to ensure adaptability in resource-limited environments.

## Introduction

Emergency department (ED) overcrowding represents a critical global challenge, exacerbated by population growth, aging demographics, and unpredictable disease outbreaks (1). This high demand often surpasses available resources, leading to prolonged wait times and increased inpatient mortality (2, 3). The situation is particularly acute in low- and middle-income countries, where pre-existing shortages of personnel and equipment intensify systemic inefficiencies (3, 4). Triage systems are designed to prioritize patient care when resources are scarce. Standard practice involves a clinician assigning an initial urgency level, typically using a four- or five-tier scale, based on the patient's presenting complaints (4, 5). However, complex systems like the Emergency Severity Index (ESI) can be challenging to implement effectively in low-resource settings, underscoring the need for more pragmatic tools (4). Furthermore, research has identified a fundamental limitation of many triage instruments: poor sensitivity for identifying critical, time-sensitive illnesses (6).

Conventional triage is inherently static. The assigned category is a snapshot from a single moment and is seldom updated as the patient's condition evolves (5). This creates a vulnerability where clinical deterioration after the initial assessment may go unrecognized. The COVID-19 pandemic revealed the consequences of this inflexibility, as static systems struggled to adapt to a novel disease with changing symptomatic presentations (1).

In this context, artificial intelligence (AI) and machine learning (ML) present a promising avenue for innovation. These technologies can continuously analyze diverse data streams, including vital signs, laboratory results, and clinical notes, to dynamically predict patient acuity and flag those at risk of clinical decline (7, 8). Recent evidence suggests that AI and ML-enhanced triage systems can improve emergency care prioritization by integrating structured clinical variables with unstructured data such as free-text symptoms, dispatch transcripts, and clinician observations (9). Natural language processing (NLP) techniques have demonstrated the ability to transform patient-reported symptoms into actionable triage information, enabling more responsive and accurate patient risk stratification. Importantly, current literature emphasizes that patient acuity is dynamic rather than static, highlighting the importance of repeated reassessment and re-triage during emergency department stays (9). This is particularly relevant in overcrowded and low-resource emergency settings, where delayed recognition of clinical deterioration contributes to preventable morbidity and mortality. AI-driven dynamic triage systems may therefore support continuous risk reassessment, reduce variability in decision-making, and optimize limited healthcare resources (9).

Early evidence suggests that ML-driven triage models can outperform traditional methods, demonstrating superior discrimination for high-risk patients and more accurate prediction of resource needs (8, 10). Such dynamic systems could enhance efficiency in busy EDs by automating data integration and supporting clinical decision-making (7).

A significant gap persists in triage practices for low-resource EDs: the absence of real-time, adaptive reassessment. Once an initial triage level is assigned, no automated mechanism exists to revise this risk score with new clinical information. Consequently, patients whose conditions worsen in the waiting area may not be identified until they reach a critical state. Studies indicate that under-triage is a common problem, occurring in approximately 25%–30% of cases in some environments (11). The implications are serious; under-triaged patients experience delays in necessary interventions, which are independently associated with higher rates of clinical deterioration, morbidity, and mortality (12, 13).

By enabling continuous risk reassessment, an AI-driven triage system could ensure the timely identification and treatment of deteriorating patients, thereby potentially reducing morbidity and mortality in overcrowded EDs. International evidence indicates that the implementation of structured triage alone can lower patient mortality (14); an AI-enhanced dynamic model could build significantly upon these benefits. Furthermore, by improving prioritization accuracy and consistency, the system could alleviate cognitive burden on clinicians, allowing them to focus on the most critical cases. Given the established link between ED crowding and adverse patient outcomes (2), any intervention that mitigates workflow bottlenecks holds substantial promise. Prior research supports the potential of ML models to enhance predictive accuracy in emergency care (10). If successfully validated and implemented, the proposed system could optimize the use of limited medical resources and improve emergency care delivery in challenging settings. This study aims to evaluate the potential of AI-driven dynamic triage systems for continuous patient risk reassessment in low-resource emergency departments, assessing their impact on patient outcomes, workflow efficiency, and resource allocation.

By enabling continuous risk reassessment, an AI-driven triage system could ensure the timely identification and treatment of deteriorating patients, thereby potentially reducing morbidity and mortality in overcrowded EDs. International evidence indicates that the implementation of structured triage alone can lower patient mortality (14); an AI-enhanced dynamic model could build significantly upon these benefits. Furthermore, by improving prioritization accuracy and consistency, the system could alleviate cognitive burden on clinicians, allowing them to focus on the most critical cases.

## Methodology

This work represents a narrative review of publications spanning 2014–2025. The search spanned multiple databases, including PubMed, Google Scholar, Web of Science, and ResearchGate, and the search strategy included the following terms: (“Triage system” OR “Emergency triage”) AND (“Artificial Intelligence” AND “Machine Learning”) AND (“LMIC” OR “Low resource countries”). The included studies were cohort, RCT, and cross-sectional designs. Editorials, commentaries, and non-English publications were excluded to maintain focus and clarity.

The population involved both adult and pediatric emergency patients in LMICs and excluded all non-emergency patients or emergency patients from well-developed countries. The intervention was any use of Artificial Intelligence, and the outcomes involved mortality and ICU admissions. Two reviewers (MJ and AS) were involved in screening and extracted relevant information. Rayyan was used for screening. Any disagreements were addressed with a third reviewer (UB). Information such as authors, study design, manuscript type, publication year, study setting, sample size, and population characteristics was noted. Artificial intelligence usage or any other techniques used were recorded, along with outcomes such as ICU admission and mortality. Studies were included if they addressed triage systems, AI or ML applications in emergency care, or challenges specific to LMICs.

**Figure 1:**
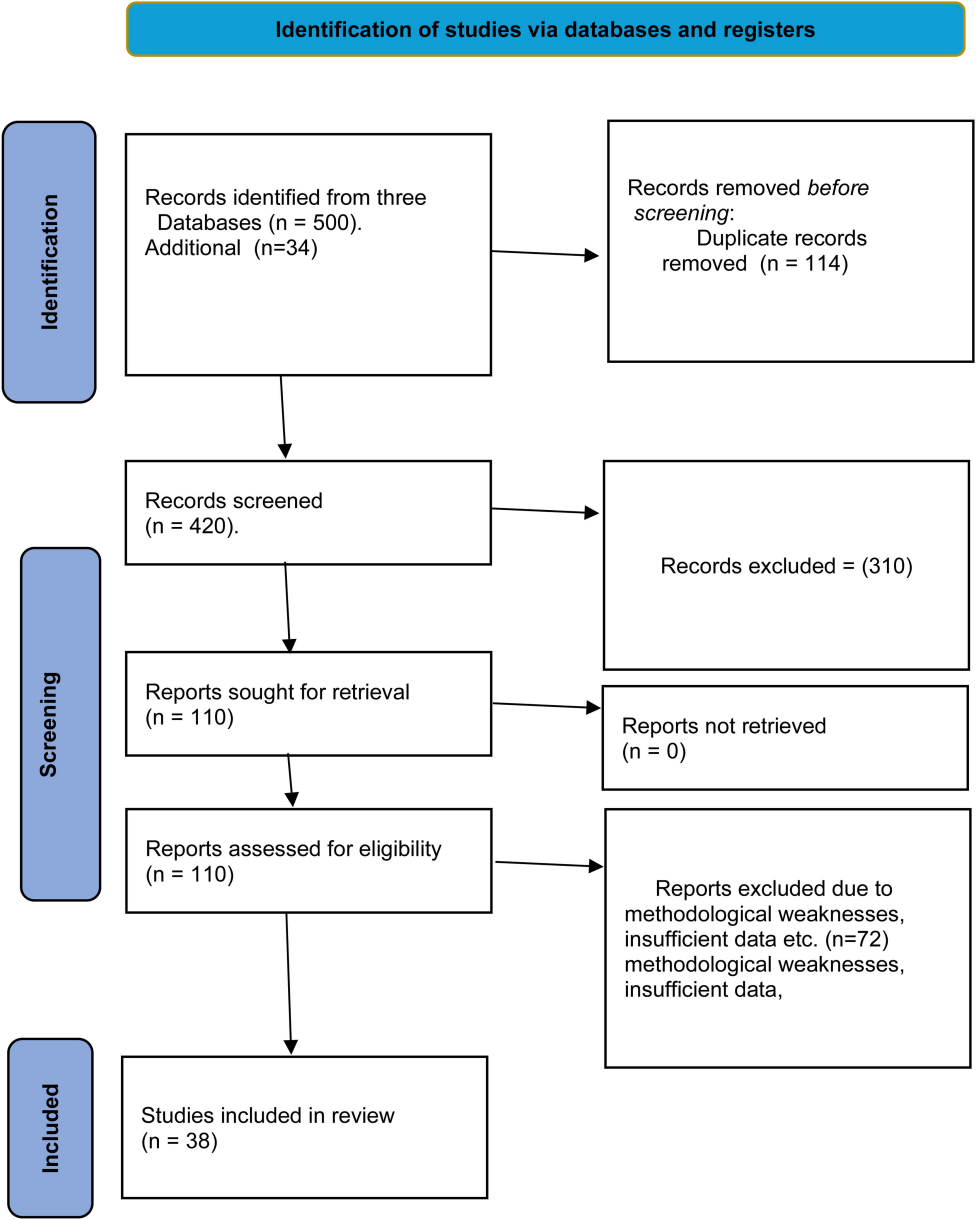
PRISMA chart.

The screening process began with titles and abstracts, followed by full-text review of relevant articles. The search initially identified just over 500 records, with an additional 34 articles retrieved through reference lists. After removing duplicates, approximately 420 unique studies remained for title and abstract screening. Of these, 110 full-text articles were assessed in detail, and 72 were excluded due to methodological weaknesses, insufficient data, or lack of relevance to low-resource emergency triage. Ultimately, 38 studies were included in the qualitative synthesis, and 24 provided sufficient data for comparative analysis. This study selection process followed PRISMA principles to improve transparency in evidence identification and screening. See Figure 1.

Findings were synthesized narratively, with quantitative summaries applied where appropriate. This approach allowed us to compare static vs. dynamic triage systems, highlight gaps in existing research, and assess the theoretical impact of AI-driven models on patient outcomes, workflow efficiency, and resource allocation. By weaving together evidence from both high-income and low-resource settings, the review aimed to provide a holistic perspective on how dynamic triage could reshape emergency care delivery.

## Results

### Overview of triage systems in emergency medicine

Triage refers to the classification of patients according to how urgent their medical needs are. This approach makes sure that critically ill patients receive urgent care (1). It plays an essential role in emergency medicine by enhancing patient flow and resource utilization. This process helps reduce delays and overcrowding in emergency departments (1). Across the globe, standardized triage models such as Emergency Severity Index (ESI), Canadian Triage and Acuity Scale (CTAS), Manchester Triage System (MTS), and Australasian Triage Scale (ATS) classify patients into systems with three or five levels. The five-level models tend to provide better accuracy and reliability in clinical settings (1). A 2025 meta-analysis of 510,777 patients showed that the ESI has moderate-to-high diagnostic accuracy for identifying critically ill adults. It reported a pooled sensitivity of 81.8% and a specificity ranging from 70.5% to 81.7% for predicting short-term mortality and ICU admissions (1). In pediatric care, organized systems such as PaedCTAS, MTS, and ESI version 4 have proven to be reliable and valid in predicting hospital admissions and ICU transfers (2). Additionally, alert-based tools like PEWS, POPS, and PAT help in the early detection of clinical deterioration (2). Overall, evidence supports the effectiveness of both adult and pediatric. However, ongoing validation, cultural translation, and staff education are necessary to ensure that these systems function effectively globally.

### Current models of emergency triage

No triage model achieves universal accuracy. However, models like Emergency Severity Index (ESI), Canadian Triage and Acuity Scale (CTAS), Manchester Triage System (MTS), and South African Triage Scale (SATS) are the best validated emergency triage systems (3). The ESI uses a five-level system that combines clinical acuity with predicted resource needs, making it efficient in busy emergency departments (3). The CTAS, which also has five levels, categorizes patients according to symptom severity and clinical modifiers for accurate prioritization in complicated presentations (3).

The MTS uses structured, color-coded flowcharts based on presenting complaints, which promotes consistency in decisions made by nurses (3). In contrast, the SATS includes physiological measurements and clinical indicators, making it suitable for low-resource settings that handle a high number of patients (3). Additionally, the TRIAGE trial showed that implementing MTS-based triage integration between emergency and general departments improved patient flow, reduced unnecessary emergency admissions, and sustained safe results (3). Overall, these findings highlight that structured triage models, when tailored to specific contexts, improve the efficiency of emergency care healthcare systems (Table 1).

**Table 1:** Diagnostic accuracy of conventional triage systems.

Triage System	Levels	Key Features	Reported Performance	Notes
Emergency Severity Index (ESI)	5	Combines acuity + resource prediction	Sensitivity: 81.8%; Specificity: 70.5–81.7 (meta-analysis, 510,777 patients)	Widely used in high-income EDs; less effective in LMICs
Canadian Triage and Acuity Scale (CTAS)	5	Symptom severity + clinical modifiers	Reliable for complex presentations	Requires strong diagnostic support
Manchester Triage System (MTS)	5 (color-coded flowcharts)	Structured decision support	Improved patient flow in TRIAGE trial	Nurse-led; consistency depends on training
South African Triage Scale (SATS)	4	Physiological measures + clinical indicators	Suitable for high-volume, low-resource EDs	More adaptable to LMIC contexts
Pediatric Systems (PaedCTAS)	3–5	Pediatric-specific adaptations	Reliable for predicting admissions/ICU transfers	Requires ongoing validation

### Limitations of conventional triage in low-resource settings

Traditional triage systems such as ESI, CTAS, MTS, and SATS have been developed for high-income emergency departments and prove to be poor performers in low-resource settings (5). Their effectiveness decreases due to variations in patient flow, disease conditions, and scarce diagnostic facilities (5). These systems are static; once a triage category is assigned, it is rarely updated, even if the patient's condition worsens (6).

This inflexibility is a reason for under-triage and delayed treatment of critically ill patients (6). In LMICs, the accuracy of nurse-led triage relies heavily on clinical experience and training, resulting in variable outcomes across facilities (5). Some sub-Saharan African hospitals have under-triage rates as high as 30%, indicating significant patient-safety risks (5). The reliance on tools like pulse oximeters and lab tests limits their use in resource-strapped environments (5). Incomplete documentation further reduces reliability, with vital signs recorded for fewer than 25% of pediatric patients in some LMIC hospitals (5).

Additionally, these triage models do not incorporate ongoing physiological data for continuous reassessment (6). As a result, traditional triage systems continue to be inconsistent, inflexible, and poorly suited to LMIC emergency departments (6) (Table 2).

**Table 2:** Limitations of conventional triage in low-resource settings.

Limitation	Evidence	Impact
Static categorization	Rarely updated after initial assessment	Misses deterioration; delays intervention
Under-triage	Rates up to 25%–30% in LMICs	Higher morbidity and mortality
Reliance on diagnostics	Pulse oximeters and labs often unavailable	Limits the applicability in resource-strapped EDs
Documentation gaps	Vital signs are recorded in <25% of pediatric cases	Reduces reliability and safety
Training variability	Nurse-led triage depends on experience	Inconsistent outcomes across facilities

### Role of artificial intelligence in clinical decision support

AI has become a significant force in modern healthcare. It is greatly improving clinical decision support systems (CDSS) through enhanced diagnostic accuracy, operational effectiveness, and patient outcomes (7, 8). In emergency care and managing syncope, AI-driven CDSS with ML, deep learning (DL), and NLP can analyze complex data to find subtle patterns that clinicians often overlook (8). These systems help differentiate true syncope from other temporary loss of consciousness, estimate short-term adverse outcomes, and estimate how long a person will need to stay in the hospital (8).

In emergency and triage care, AI algorithms are more predictive in accuracy than conventional means in measuring patient deterioration, disease severity, and need for intervention. This improves clinical decision-making (7). A primary strength of AI-based triage systems is their capacity to improve the speed, consistency, and accuracy of patient prioritization through real-time clinical data analysis (15). Authors emphasize the specific role of machine learning and NLP in integrating structured data, such as vital signs, with unstructured clinical information, including patient complaints and physician notes. AI-assisted triage systems could support more efficient allocation of limited healthcare resources and facilitate earlier recognition of clinical deterioration (15). When combined with electronic medical records (EMRs), AI-based CDSSs can offer personalized diagnostics and treatment suggestions, reduce prescription errors, improve adherence to guidelines, and cut down on unnecessary tests. This ultimately lowers healthcare costs (7, 8). Additionally, wearable sensors, along with AI algorithms, provide real-time monitoring of vital signs. This helps with early detection of arrhythmias, vasovagal events, or hemodynamic instability (8). However, AI's advantage over doctors is still unclear due to issues with data quality, lack of outside validation, and ethical concerns (7, 8). However, AI-enabled CDSSs represent an important advancement towards medicine by complementing doctors' expertise with predictive, data-driven intelligence (8).

### Machine learning models used in healthcare risk prediction

ML has become increasingly popular in healthcare, particularly for tasks involving prediction, such as identifying disease, estimating outcomes, and guiding clinical decisions. These models are capable of detecting subtle and nonlinear patterns in large, complex datasets. Such patterns often go unnoticed when using conventional statistical approaches (1). In emergency care, these techniques have been used to predict several critical outcomes. For example, they help anticipate patient decline, estimate the likelihood of death, detect early signs of sepsis, and assess the risk of hospital readmission. Many of these predictions can be made in real time, allowing clinicians to respond more quickly and effectively (2).

Among the most commonly used ML models for healthcare risk prediction are logistic regression (LR), random forest (RF), gradient boosting machines (GBM), and support vector machines (SVM). In more complex applications, researchers also use convolutional neural networks (CNNs) and recurrent neural networks (RNNs) to analyze patterns and predict clinical outcomes (3).

Despite its simplicity, logistic regression continues to play an important role in clinical risk modeling. Its structure allows for clear interpretation of results, which is especially useful when working with limited data or when transparency is essential for clinical review and validation (4, 5). In large datasets, models like random forest and gradient boosting often perform better than simpler approaches. They work by combining the results of many smaller models, which helps reduce the risk of overfitting and leads to more accurate predictions overall (6, 7).

Recent modeling techniques have made it easier to work with time-based and mixed types of clinical data. One approach, long short-term memory (LSTM) networks, has been especially useful for tracking changes in patient status over time. These models can process sequences like vital signs, lab results, and continuous monitoring data to help spot early signs of physical decline, often before they become critical (8, 10). For example, a study showed that models built using LSTM techniques were more effective than conventional scoring systems when it came to predicting in-hospital death among patients in emergency settings (11). Similarly, transformer-based models such as Bidirectional Encoder Representations from Transformers (BERT) have been used to analyze unstructured text in electronic health records. These models help extract important risk-related information from patient notes and other free-text entries. By automating this process, they reduce the need for manual review and make it easier to identify relevant clinical features (12, 13).

In places where resources are limited, using advanced models can be challenging. This is often due to patchy data and unreliable digital systems. Still, simpler approaches, like logistic regression or shallow neural networks, can be practical options for supporting triage decisions in real time. When paired with cloud services or edge computing, these models can run efficiently without needing heavy infrastructure, making them more accessible in remote or underserved areas (14, 16, 17).

When using predictive models in clinical triage, one of the most important factors is whether the model's decisions can be clearly understood. Tools like SHAP and LIME have become more common because they help explain how a model arrives at its conclusions. By showing which factors influenced a prediction, these tools make it easier for clinicians to trust and validate what the model is telling them (18). Bringing predictive models into triage systems means patient risk can be reassessed continuously, not just at intake. This ongoing evaluation helps emergency departments stay responsive, moving patients through more efficiently and using staff and resources where they're needed most (Table 3).

**Table 3:** Machine learning models in healthcare risk prediction.

Model	Strengths	Limitations	Example Applications
Logistic regression (LR)	Transparent, interpretable	Less accurate in large datasets	Mortality prediction, sepsis risk
Random forest (RF)	Handles nonlinear data, reduces overfitting	Computationally heavier	ICU admission prediction
Gradient boosting machines (GBM)	High accuracy, ensemble approach	Less interpretable	Hospital readmission risk
Support vector machines (SVM)	Effective in small datasets	Limited scalability	Disease classification
LSTM networks	Captures time-series changes	Needs large datasets	Predicting deterioration via vital signs
Transformer models (e.g., BERT)	Analyzes unstructured text	Complex, resource-intensive	Extracting risk factors from EHR notes

### Dynamic vs. static triage: a comparative perspective

Most traditional triage systems, like the Emergency Severity Index (ESI), Canadian Triage and Acuity Scale (CTAS), and Manchester Triage System (MTS), work in a fixed way. They assign a patient's urgency level right when they arrive, and that level usually doesn't change, even if the patient's condition does. These systems depend a lot on the clinician's first impression and how they interpret the symptoms, which can be quite subjective (19, 20). Standard triage systems may follow a fixed protocol, but they often miss when a patient's condition starts to deteriorate quickly. This is a real concern in overcrowded emergency departments, where time is critical and clinical situations can shift in minutes (21).

Unlike fixed triage models, dynamic systems are designed to respond to how a patient's condition evolves. They don't rely on a single assessment at arrival; instead, they reassess the patient regularly, either continuously or at set intervals, using up-to-date clinical information. Some of these systems go a step further by pulling in real-time data like heart rate, oxygen saturation, and breathing patterns, along with notes from clinicians. Rather than sticking to one risk level, they update it throughout the patient's stay in the emergency department, helping guide decisions based on the most current picture of the patient's health (22, 23).

This reflects a major shift in how triage is being approached. Instead of treating patient prioritization as a one-time decision made at arrival, the focus is now on adjusting as the patient's condition changes. Benson et al. pioneered this approach with a methodology allowing triage to evolve over hours or days, maximizing patient survival (24) (Table 4).

**Table 4:** Static vs. dynamic triage systems.

Feature	Static Triage	Dynamic Triage
Assessment	One-time at arrival	Continuous or interval reassessment
Data inputs	Presenting complaints, initial vitals	Real-time vitals, labs, and clinician notes
Adaptability	Limited	Updates risk level as condition evolves
Bias	Higher (subjective, first impression)	Reduced (data-driven, adaptive)
Suitability in LMICs	Poor (requires diagnostics, documentation)	Potentially better with mobile/cloud AI

Dynamic triage also facilitates risk stratification equity. Traditional systems often miss the subtle differences in how patients present, especially when it comes to age, gender, or existing health conditions. These blind spots can lead to biased decisions and unequal care (22, 24). Some systems powered by machine learning can adjust in real time, using data that reflects the unique characteristics of different patient groups (25). This helps reduce bias that often goes unnoticed in traditional triage models. But putting these systems into practice isn't easy. They rely on strong digital infrastructure and the ability to pull together data from multiple sources (26). In places where resources are limited, building and maintaining that kind of setup is a major challenge.

### AI applications in emergency medicine

Emergency medicine has seen rapid growth in the use of artificial intelligence over the past five years. These tools are now being used in areas like diagnostic imaging, early detection of sepsis, managing patient flow, and automating triage decisions. In well-resourced healthcare systems, the impact has been clear: clinics are running more efficiently, and patients are receiving better care. For example, voice-based documentation tools have helped speed up charting by nearly 20%, and ML models have made triage more accurate, cutting down errors in patient prioritization by as much as 9% (27, 28). A study from South Korea in 2021 tested a new approach to triage by combining natural language processing with electronic health records. The system automatically pulled out key information, like patients' main complaints and patterns in their vital signs, and used that to predict ICU admissions. Compared to manual triage, this method proved more accurate and responsive (29).

Across the Global South, small-scale pilot programs are starting, even in places where infrastructure is limited. These early efforts reflect a growing commitment to finding workable solutions that fit the realities of local healthcare systems. In 2025, researchers developed a simple symptom triage tool built for low-cost smartphones, designed with support for local languages. It was created with rural communities in mind, making it easier for people in underserved areas to access basic healthcare guidance. Their work shows how technology can be shaped to fit the realities of the setting, rather than forcing a one-size-fits-all solution (30). Hussain and colleagues similarly highlighted the tough challenges that come with using digital health tools in low-resource environments, especially when it comes to cost and infrastructure. Still, they pointed to the real potential these tools have in closing health gaps and reaching people who’ve long been left out of the system (31).

Recent studies confirm that AI in emergency medicine has the potential to enhance decision accuracy, resource allocation, and patient safety (32, 33). One of the biggest challenges is that these systems don’t always translate well across different settings. Variations in data quality, patient demographics, and how emergency departments are set up from place to place make it hard to apply findings more broadly. On top of that, most AI tools in emergency care are still in the testing phase. Very few have been fully integrated into everyday clinical practice (34).

### Challenges of implementing AI in low-resource health systems

Delivering emergency care in low-resource settings has complex challenges. These include gaps in technology, weak infrastructure, ethical concerns, and unclear regulatory pathways, all of which make implementation difficult. One of the biggest hurdles is the lack of reliable health data. Many healthcare systems in low- and middle-income countries still operate without standardized electronic records. This absence makes it hard to build dependable tools that rely on consistent information. Even when records exist, they're often incomplete or entered inconsistently. Missing details and disconnected systems further weaken the foundation needed for effective decision-making and care delivery (16, 35, 36). Implementation challenges in low-resource settings include limited digital infrastructure, fragmented health records, concerns regarding data quality, and ethical considerations (15).

Hospitals in low-resource settings often struggle to meet the basic requirements needed to support modern digital tools. Consistent electricity, high-capacity computing systems, and stable internet connections are frequently unavailable. These gaps make it difficult to run systems that rely on continuous power and real-time data exchange (35, 37, 38). Even when partial solutions like cloud platforms or mobile health tools are introduced, they often come with compromises. Relying on external servers can raise concerns about data privacy, slow response times, and long-term sustainability, especially in underfunded institutions (39, 40).

Another major hurdle is the shortage of skilled personnel. Effective implementation depends on collaboration between clinicians, technical staff, and data specialists. Yet in many developing countries, such multidisciplinary teams are rare (37, 38, 41). Most healthcare workers have limited exposure to digital systems, and those in rural areas often receive little to no formal training. This makes intuitive design and tailored education essential for any new tool to be usable and effective (38, 42).

Finally, sustainability is a critical concern. Even when low-cost or open-access tools are available, maintaining them requires ongoing investment in updates, cybersecurity, and technical support resources that many institutions simply don't have. If not carefully managed, the push for digital innovation could divert attention and funding away from essential clinical services, potentially deepening existing health inequities (43).

In many low-resource settings, the lack of formal oversight makes it difficult to introduce digital tools in a safe and accountable way. Without clear policies or regulatory guidance, there is a greater risk of poor decision-making, limited transparency, and inadequate protection of patient rights, especially when it comes to informed consent (37, 41, 44). These concerns are amplified when technologies designed in high-income countries are brought in without being adapted to local needs. Such systems may fail to reflect the cultural, clinical, or infrastructural realities on the ground. This raises important questions about who controls the data, how decisions are made, and whether communities have a say in the tools being used (41, 43, 45).

### Gaps in existing research and the need for dynamic AI triage models

Most existing research centers on fixed, one-time prediction models based on past data. Few systems are designed to update patient risk assessments continuously in real time an especially important gap in emergency departments and resource-limited settings (23, 46–48).

A critical synthesis of the 38 included studies reveals a significant tension between the theoretical capabilities of AI-driven dynamic triage and the practical hurdles of real-world implementation, particularly in low-resource settings. The following synthesis evaluates the evidence while identifying specific biases and limitations associated with key studies.

1. Methodological and retrospective bias

A major limitation across the evidence base is the heavy reliance on past data rather than live testing. Yi et al. (49) and Siira et al. (47) highlight a “retrospective bias,” where AI models show high predictive accuracy in historical simulations but lack sufficient prospective validation in real-world emergency departments (4–6, 47–49). Furthermore, the study by Porto (23) indicates that while ML can improve triage performance, the majority of research focuses on model development rather than clinical integration or patient outcomes (21–23). The authors of the review itself acknowledge a potential selection bias, as the study was conducted as a narrative review rather than a strict systematic review.

(1) Comparative synthesis: conventional vs. dynamic models

Traditional triage systems are criticized for being “static snapshots” that fail to account for patient deterioration.

- Subjective Bias: Storm-Versloot et al. (20) and Hinson et al. (21) identify that conventional systems like the Emergency Severity Index (ESI) and Manchester Triage System (MTS) rely heavily on subjective clinician impressions, which can lead to inconsistencies (19–23).
- Performance Gaps: While ESI maintains high sensitivity for mortality, its effectiveness is significantly lower in low- and middle-income countries (LMICs) due to limited diagnostic resources (20).
- Dynamic Innovation: Benson et al. (24) pioneered the “dynamic triage” concept, which is now being realized through AI models that continuously update risk scores. Chen et al. (22) demonstrate the superiority of this approach by integrating real-time ECG and CXR data to identify high-risk patients who might be missed by static arrival-only assessments (21–23).
- Model complexity vs. transparency

The synthesis of machine learning architectures reveals a trade-off between accuracy and explainability.

- Interpretability vs. Accuracy: Hua et al. (4) and Arjunan (18) advocate for Logistic Regression (LR) because its transparency is vital for clinical trust, even though it is less accurate for complex datasets (4, 18).
- The “Black Box” Problem: Conversely, Deng et al. (11) and Shashikumar et al. (10) find that Deep Learning (LSTM) and Ensemble models (Random Forest, Gradient Boosting) offer superior accuracy for mortality and sepsis prediction but are more computationally intensive and harder for clinicians to interpret (7–13)^.^
- Geographic, demographic, and infrastructure biases

A critical flaw in current AI research is the lack of generalizability to non-Western settings.

- Algorithmic Bias: Yang et al. (51) explicitly identify an ML bias between high-income countries and LMICs, noting that models trained on Western demographics often fail to perform accurately in the Global South due to different disease patterns (50–52).
- Demographic Underrepresentation: Di Sarno et al. (52) and Yu & Zhai (45) point out a significant “demographic bias” where pediatric and obstetric populations are underrepresented in training data, raising concerns about fairness and inclusivity (44–46, 50–52).
- Infrastructure Bias: Hussain et al. (31) and López et al. (37) argue that many “innovative” digital tools are biased toward high-resource environments, assuming stable electricity and internet that are often absent in LMICs (16, 30–32, 36, 37).
- Human factors and implementation hurdles

Finally, the synthesis highlights that technical success does not equate to clinical success.

- Trust Bias: Townsend et al. (54) researched practitioner perspectives, finding that a lack of clinician trust and “human-in-the-loop” design are major barriers to AI adoption (53, 54).
- Sustainability Bias: Joshi et al. (43) note that many digital health interventions fail because they ignore the long-term costs of maintenance, cybersecurity, and staff training in developing nations (41–43).

A few recent studies have started exploring more flexible or follow-up screening approaches, but these efforts are still in early stages and haven't yet gained widespread acceptance or validation (22, 48).

Most published studies still rely heavily on retrospective data. Forward-looking research remains limited, particularly studies evaluating real-world impact. There's a particular gap in evidence regarding patient outcomes, workflow efficiency, and the integration of these tools into everyday clinical practice (23, 47, 49, 50). These limits understanding of how AI triage affects actual care delivery.

Models built in high-income countries often don't perform as well when used in lower-income settings. Differences in patient demographics, disease patterns, and how people seek medical care can significantly affect how these tools work. Without thoughtful adjustments, their accuracy and usefulness in new environments remain limited (35, 51, 52). When certain groups, like pediatric or obstetric cases, are underrepresented in the data, it becomes harder to apply findings broadly. This imbalance not only weakens the reliability of the models but also raises concerns about fairness and inclusivity in care delivery (53).

Many hospitals, particularly in LMICs, face serious challenges when trying to connect advanced triage systems with their existing infrastructure. The cost of integration and the technical complexity involved often make it unfeasible. At the same time, there's a noticeable lack of research into simpler, more adaptable triage tools, especially those designed to run on mobile phones or small devices. These kinds of solutions could make a big difference in scaling emergency care, but they're still largely missing from the conversation (35, 48). The way healthcare professionals interact with technology and whether they trust it hasn't been studied enough. That's a problem, because these factors play a huge role in whether new tools are used safely and effectively. Equally important is involving clinicians and other end-users in the design process from the start. Without their input, it's hard to build systems that truly support clinical decision-making rather than disrupt it (47, 52, 54).

## Discussion

### Interpretation of findings

The shortcomings of conventional triage systems, such as the MTS, ESI, and CTAS, in demonstrating effectiveness in LMICs have been well documented. These shortcomings are attributed to varying patient demographics, limited diagnostic facilities, and differing disease trajectories (1–4). The studies reviewed do not adequately explore the untapped potential of dynamic triage systems using AI to track patient risk, utilizing physiological data and clinical reports to improve disease progression or outcomes (5–7). Traditional static triage strategies have been shown to fail in capturing the deterioration of patient health, leading to delays in providing quality care (3, 4).

This present study analyzed that random forests, gradient boosting machines, logistic regression, LSTM networks, and transformer-powered architectures can effectively uncover non-linear, complex data, thereby proving superior to traditional techniques (5–8). Coupling these systems with digital health records and wearable devices is a proposed plan to incorporate real-time patient information, improving care delivery, and ending the reliance on outdated static triage methods (1, 2, 7, 8).

### Implications for emergency department management

The introduction of AI-powered dynamic triage systems in emergency departments is expected to improve emergency care outcomes. Firstly, continuous, 24/7 risk assessment enables the early detection of severely ill patients, which in turn reduces the strain on healthcare departments and lowers both mortality and morbidity (5, 6). According to the established evidence, an AI-driven dynamic triage system should comprise inputs of vitals like heart rate, respiratory rate, blood pressure, and SpO_2_, along with all related clinician comments, supplementary reports, consecutive lab results, and initial triage scores. A standard threshold for each vital sign should be assigned, which, if reached, should alert the healthcare staff or physician immediately. This approach looks like it is already being used, but assisting it with a notification system showing the probability of deterioration will help direct attention to the high-risk potential deteriorating health patients, and that would mean a reassessment every 15–30 min of these patients should be done. To evaluate the efficacy and success rates of this approach, a validation or experimental study can be done using this approach in LMIC countries using the same cohort of patients assessed in other systems, and the criteria should involve ICU admission and mortality. Secondly, clinician burnout can be mitigated by standardizing decision-making, reducing interobserver variability, and automating operations (7, 8). Thirdly, resource allocation can be optimized by prioritizing diagnostic and therapeutic interventions based on patient condition (1, 3, 7).

Most importantly, AI programs will enhance efficacy in pediatric and vulnerable populations by addressing gaps in the Pediatric Early Warning Score (PEWS), Pediatric Observation Priority Score (POPS), and Pediatric Assessment Triangle (PAT) (2). This suggests that the new, improved AI-driven dynamic triage system may offer numerous benefits, such as smoother patient flow, better utilization of resources on evidence-based protocols, reduced overcrowding, and ultimately enhanced efficiency (1, 3).

### AI as a tool for health system strengthening in LMICs

AI-based triage and predictive models have immense potential to strengthen emergency care in low- and middle-income countries (LMICs) by providing economical, versatile, and real-time clinical decision support. AI standardizes care, ensures equitable prioritization, and improves outcomes (11–13). Less modernized settings with poorer infrastructure particularly benefit, as mobile and cloud-based AI platforms enable continuous patient monitoring in these environments (11). Moreover, AI systems offer training and clinical guidance to less experienced staff, allowing for evidence-based practices in settings with limited access to specialists (11, 12).

Furthermore, AI integration strengthens the health system by collecting structured data for epidemiological studies, identifying systemic bottlenecks, and guiding the distribution of resources, which is crucial for healthcare policy and strategy in LMICs (11, 13)^.^

### Potential barriers to adoption

Despite the potential benefits AI offers, several constraints may limit its adoption in emergency care settings in LMICs. First, infrastructure limitations such as unstable electricity, low computational capacity, and poor internet connections hinder the use of real-time AI (13–15). Second, there are significant data deficiencies. Since electronic medical records are fragmented and far from standardized, the resulting AI models often suffer from inaccuracies and lack generalizability (3, 4, 14). Third, human factors pose another barrier: inadequate staff training in AI tools and resistance to adopting advanced technologies may hinder effective integration (4, 14, 15). Fourth, ethical concerns limit AI usage, including biases in data patterns, confidentiality issues, consent policies, and the need for strong oversight (11, 14, 15). Lastly, sustainability is a major concern due to the capital and expertise required for maintenance, updates, and cybersecurity (13–15).

### Policy and implementation recommendations

To strengthen AI adoption, LMICs must upgrade infrastructure by improving digital computing capacity through better internet connectivity, a stable and uninterrupted electricity supply, and either on-premises local storage or cloud-based computing and storage solutions (11, 12, 15). Standardized data practices are also essential; national or regional standards for e-health records and clinical documents should be established to refine and enhance the performance and capabilities of AI (3, 4). Workforce development is equally important, requiring regular training programs for clinicians, nurses, and data specialists to ensure the efficient use of AI systems (4, 14). Ethical governance and regulatory structures must be implemented to protect patient data, reduce bias in clinical decision-making, and ensure algorithmic transparency (11, 14, 15). Pilot implementation and local adaptation should involve identifying locally available mid- to low-tier AI tools that fit the clinical and cultural context (13). Additionally, workflow integration is essential; instead of replacing human judgment, AI systems should be designed and implemented alongside clinicians to maintain accountability and proper supervision (5–7). By acknowledging and implementing these recommendations, LMIC healthcare systems can integrate and utilize AI to increase accuracy, improve patient outcomes, and enhance emergency care logistics, ultimately reducing the gap between resource availability and health equity (1, 3, 5–7, 13).

While dynamic AI triage represents a “transformative opportunity”, the field is currently limited by retrospective methodologies and geographic biases. Moving forward, research must shift toward **prospective, locally adapted studies** that prioritize vulnerable populations and ensure that AI complements, rather than replaces, human judgment in the emergency department.

### Study limitations

This study has several limitations that should be acknowledged. As a narrative review, the synthesis followed a structured evidence identification and selection process, but variability in included study designs may still introduce selection bias. Much of the available evidence is derived from retrospective studies and pilot programs, limiting the ability to assess the real-world impact of AI-driven triage systems. The heterogeneity of study settings, ranging from high-income hospitals with advanced infrastructure to low-resource emergency departments, makes direct comparisons difficult and may affect generalizability. In addition, the underrepresentation of pediatric, obstetric, and LMIC populations constrains the applicability of findings to these vulnerable groups. Finally, issues of data quality, incomplete documentation, and lack of external validation remain significant barriers.

Future research should therefore prioritize prospective validation studies of AI-driven dynamic triage systems in real-world emergency departments, particularly in LMICs. These studies must evaluate direct impacts on patient outcomes, workflow efficiency, and resource allocation. Greater emphasis should be placed on equity and inclusivity, ensuring that pediatric and obstetric populations are adequately represented. Research should also explore mobile-first and cloud-based solutions that can function in environments with limited infrastructure, making dynamic triage accessible in rural and underserved areas. Another critical direction is co-design with clinicians and end-users, ensuring that AI tools are intuitive, trusted, and integrated seamlessly into existing workflows. Finally, future studies should investigate ethical and regulatory frameworks for AI in emergency care, focusing on transparency, data governance, and patient rights to build sustainable and accountable systems.

## Conclusion

AI-driven dynamic triage systems represent a transformative opportunity to strengthen emergency care in low-resource environments. By enabling continuous risk reassessment, these tools can reduce undertriage, improve patient flow, and optimize scarce resources. Yet, barriers such as poor infrastructure, limited data quality, and a lack of clinician engagement remain significant. Future research must prioritize prospective validation, equity in model design, and integration into existing workflows. With careful adaptation, dynamic AI triage can complement clinician expertise, mitigate overcrowding, and improve outcomes for vulnerable populations.

## Data Availability

The original contributions presented in the study are included in the article/Supplementary Material, further inquiries can be directed to the corresponding author.
